# Proteomics techniques in protein biomarker discovery

**DOI:** 10.1002/qub2.35

**Published:** 2024-03-01

**Authors:** Mahsa Babaei, Soheila Kashanian, Huang‐Teck Lee, Frances Harding

**Affiliations:** ^1^ Department of Biology Faculty of Science Razi University Kermanshah Iran; ^2^ Faculty of Chemistry Sensor and Biosensor Research Center (SBRC) Razi University Kermanshah Iran; ^3^ Nanobiotechnology Department Faculty of Innovative Science and Technology Razi University Kermanshah Iran; ^4^ Centre for Marine Bioproducts Development Flinders University Adelaide South Australia Australia; ^5^ Monash Institute of Pharmaceutical Sciences Monash University Melbourne New South Wales Australia

**Keywords:** biomarker discovery, cancer biomarker, gel‐based methods, gel‐free methods, mass spectroscopy, proteomics

## Abstract

Protein biomarkers represent specific biological activities and processes, so they have had a critical role in cancer diagnosis and medical care for more than 50 years. With the recent improvement in proteomics technologies, thousands of protein biomarker candidates have been developed for diverse disease states. Studies have used different types of samples for proteomics diagnosis. Samples were pretreated with appropriate techniques to increase the selectivity and sensitivity of the downstream analysis and purified to remove the contaminants. The purified samples were analyzed by several principal proteomics techniques to identify the specific protein. In this study, recent improvements in protein biomarker discovery, verification, and validation are investigated. Furthermore, the advantages, and disadvantages of conventional techniques, are discussed. Studies have used mass spectroscopy (MS) as a critical technique in the identification and quantification of candidate biomarkers. Nevertheless, after protein biomarker discovery, verification and validation have been required to reduce the false‐positive rate where there have been higher number of samples. Multiple reaction monitoring (MRM), parallel reaction monitoring (PRM), and selected reaction monitoring (SRM), in combination with stable isotope‐labeled internal standards, have been examined as options for biomarker verification, and enzyme‐linked immunosorbent assay (ELISA) for validation.

## 1 INTRODUCTION

Protein biomarkers, with characteristic properties that correlate with a biological state [1], have a substantial history of use within the cancer clinic, beginning with the demonstration of the action of tumor‐specific antigens in colorectal cancer more than 50 years ago [2]. Their application spans diagnosis, prognosis, selection of treatment, and monitoring of response.

Broadly, clinical biomarkers can be categorized into four groups: diagnostic, prognostic, predictive, and pharmacodynamic [2, 3]. Diagnostic biomarkers may be used as tools to differentiate candidate diagnoses. The Philadelphia chromosome (Ph) translocation, for example, that results from translocation between the Abelson murine leukemia *(ABL1)* gene on chromosome 9 with the breakpoint cluster region *(BCR)* gene on chromosome 22 [4], is associated with chronic myelogenous leukemia, or subset of acute lymphoblastic leukemia [5]. Prognostic biomarkers, similarly, are employed to form a diagnosis, to stratify malignancy [6], and to evaluate the probability of disease progression. Breast cancer genes 1 and 2 *(BRCA1/2)* mutations may be used as prognostic biomarkers to predict cancer prognosis in breast cancer [7]. Predictive biomarkers correlate the expected response of a patient to a particular treatment and can be helpful in optimal therapy determination [8]. Validated predictive biomarkers such as *PD‐L1* (programmed cell death ligand 1) expression in lung cancer, *BRAF* (human gene that encodes a protein called B‐Raf) status in melanoma, and *RAS* (proto‐oncogenes that are frequently mutated in human cancers) in colorectal cancer are now broadly used in daily clinical practice [9]. Pharmacodynamic response biomarkers measure treatment response and may assist in dose titration [10, 11]. It should be noted that biomarkers may fall into more than one listed category. For instance, mitotic arrest deficient 2‐like protein 1 can act both as a novel diagnostic tool and as a predictive biomarker in cholangiocarcinoma [12].

In this work, we present a review of the proteomics techniques and strategies that have been used for the discovery and validation of new biomarkers, using particular examples from cancer diagnosis and treatment.

## 2 PROTEOMICS WORKFLOW IN BIOMARKER DEVELOPMENT

**FIGURE 1:**
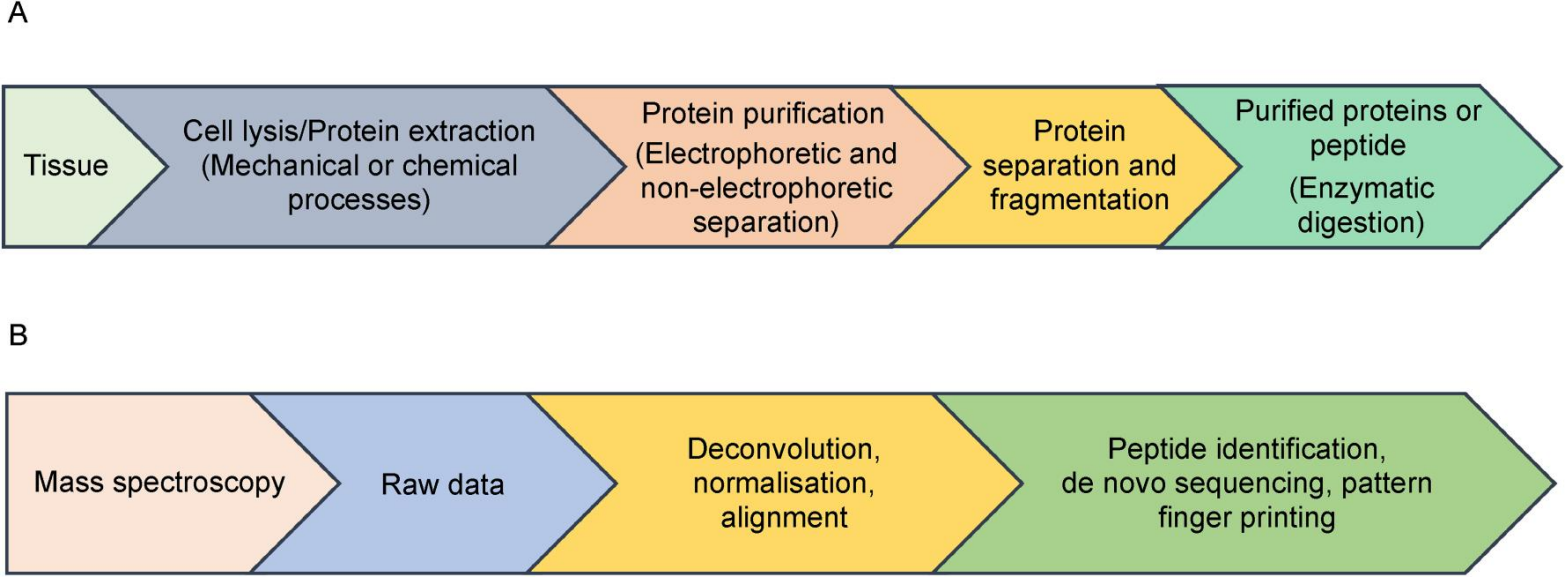
Proteomics workflow. (A) Sample preparation. (B) Attainment of protein information and database utilization [13].

The proteomics approach to biomarker identification for a specific disease comprises four steps: (1) sample preparation by extraction and separation of proteins, (2) sample analysis and data acquisition, and (3) data analysis, followed by (4) verification and validation of the biomarker (Figure 1) [14, 15].

### 2.1 Sample preparation: Extraction and separation of proteins

Various sample origins, including blood, sera, urine, cerebrospinal fluid, stool sample, and tissue biopsies can be employed for proteomic diagnosis and disease tracking [16, 17]. However, the choice of method for initial sample preparation is highly dependent on the nature of the sample and affects the selectivity and sensitivity of the downstream analysis [18]. In protein extraction, the preferred extraction method is based on the properties of the source sample, for example, liquid or solid. Therefore, if it is a solid sample that contains a large number of cells, then tissue homogenization and cell lysing should be performed. In the case of tissue samples, mechanical homogenization methods are useful. In tissue samples, mechanical homogenization methods are useful, during which physical lysing of cells can be performed by heat treatment and ultrasound; chemical methods, such as treatment with detergent solution, provide an alternative. According to the test conditions, detergents that increase the solubility of proteins can be used. In the case of liquid samples, it must also be determined whether soluble protein is required or whether the protein present in the cells must be extracted. To extract the protein from the specific cells, the protein‐containing cells must be separated by particular centrifugation. Using media of different densities may be useful for the isolation of proteins expressed in a specific cell. If a soluble protein is obtained from body fluids, it is required to be treated like solid samples [19]. Pressure cycling technology using Barocycler instrumentation, using urea as a lysis buffer, offers a novel technology that has significantly enhanced both tissue solubilization and digestion [20].

After this pretreatment some detergents and solvents used for cell lysis and protein extraction may interfere with enzymatic digestion, reverse‐phase separations, and mass spectroscopy (MS) function, and can also damage the equipment, so obtaining a purified sample is an essential step [21]. For this reason, protein purification methods using electrophoretic and non‐electrophoretic separation techniques are often employed to reduce sample complexity and to ensure assay reproducibility and accuracy [22].

#### 2.1.1 Electrophoretic separation techniques

Some of the commonly used electrophoretic separation techniques used for proteomic characterization include one‐dimensional polyacrylamide gel electrophoresis techniques (1D‐PAGE), two‐dimensional PAGE (2D‐PAGE), and difference gel electrophoresis (DIGE) [23]. If the molecular weight of the protein of interest is known, the approximate position of the band on the gel can be isolated and analyzed by MS. Advantages and disadvantages of electrophoretic separation techniques are described in Table 1.

**TABLE 1:** Advantages and disadvantages of protein separation techniques.

Separation techniques		Advantages	Disadvantages	Refs.
Electrophoretic separation techniques	1D‐PAGE	✓ Good separation; ✓ Rapid separation; ✓ Cost effective	✓ Separation is only based on the protein size; ✓ Denaturation of proteins by SDS; ✓ Not sufficient for low‐abundance, membrane, alkaline, and high molecular weight proteins preparation; ✓ Poor reproducibility; ✓ Poor resolution	[24]
	2D‐PAGE	✓ Hundreds to thousands of polypeptides and intact full‐length proteins can be analyzed in a single gel; ✓ Protein separation in the pure form from the resultant spots; ✓ Separation and identification of PTMs and protein isoforms; ✓ Easily coupled with further techniques; ✓ Higher resolution than 1D gel electrophoresis; ✓ Detection of biomarkers and disease markers	✓ Insufficient capability in the separation of hydrophobic, acidic, basic, hydrophobic, and low abundance proteins; ✓ Smaller dynamic range than some other separation techniques; ✓ Pricier than 1D‐PAGE; ✓ Low throughput and labor‐intensiveness	[24, 25]
	DIGE	✓ Higher sensitivity and reproducibility than 2‐DE; ✓ More than one sample can be run; ✓ High dynamic range of detection and quantitation; ✓ Detection and quantification of protein isoforms	✓ Proteins without lysine cannot be labeled; ✓ Special tools for visualization are required; ✓ Very expensive	[24, 26, 27]
Non‐electrophoretic separation techniques	HPLC	✓ Extremely precise; ✓ High reliability, speed, and efficiency; ✓ High sensitivity and resolution; ✓ Automated procedure; ✓ Quantitative sample recovery; ✓ Amenable to various samples	✓ Numerous expensive organics; ✓ Costly procedure; ✓ A force supply and ordinary support are required; ✓ Long test time for each sample ✓ No universal detector; ✓ More challenging for beginners	[28, 29]
	SEC	✓ Sufficient separation with a minimal eluate volume; ✓ Employing diverse solutions without interrupting the filtration procedure; ✓ Molecular weights measurement, as well as distribution of macromolecules; ✓ Multiple and nondestructive detectors; ✓ Easy‐to‐run samplings	✓ Poor reproducibility in terms of molar mass analysis, load capacity of the sample; ✓ Limited peaks can be determined because the run time is short and there are probable interactions of the solute with the solid phase; ✓ Limited range in size separation	[30, 31, 32, 33]
	Ion chromatography	✓ Investigation of a large number of molecules with high capacity, high determining ability with high levels of purification of the targeted molecule; ✓ Applied in the manufacturing scales with lower cost; ✓ Mild separation conditions; ✓ Preference to sample concentrating; ✓ Pursuing the nonsolvent extractable natural products	✓ Restricted to charged ions or polar molecules analysis; ✓ challenging to select distinct compounds for high‐speed investigation	[34, 35]

Abbreviations: PTMs, post‐translational modification; SDS, sodium dodecyl sulfate.

In 1D‐PAGE separation, protein fractionation may be achieved under native or denaturing conditions. With the former, separation is based on the respective net charge, size, and conformation of each protein molecule. The second 1D‐PAGE technique typically utilizes the anionic detergent sodium dodecyl sulfate (SDS) as a denaturant to confer a net negative charge to each protein analyte [36]. However, poor resolution and inconsistent recoveries are notable drawbacks of this method when crude unprocessed samples are used. The most important limitation is the poor recovery of proteins trapped in the gel [22, 37].

In 1975 O'Farrell developed 2D‐PAGE as a means of separating proteins electrophoretically in two dimensions; on the basis of isoelectric points in the first dimension, followed by separation by molecular mass in the second dimension [38]. This method is capable of resolving up to 10,000 proteins, including isoforms or post‐translational modifications of the same protein, as discrete spots or bands in a single gel [39]. However, this method has a number of limitations including low inter‐gel reproducibility and poor resolving power when profiling extremely hydrophobic, acidic, or basic proteins.

2D‐DIGE, which was proposed in 1997, is a modified version of 2D‐PAGE, and is employed for differential quantitative consideration of protein expression between experimental groups by utilizing differential labeling protein samples with up to three fluorescent tags [40]. The samples are analyzed based on the migration pattern, so similar proteins with individually attached dye move to the identical point on the gel. The power of this approach was demonstrated by Kirana and colleagues, who were able to identify a set of novel biomarkers to guide the treatment of colorectal cancer. Through the analysis of cancer cells from primary colorectal tumors of stage II patients, a comparison was made between a cohort with disease recurrence and a cohort that did not relapse. From this comparison, markers associated with the risk of tumor progression and with survival could be defined, allowing patients at higher risk to be identified and placed on a more aggressive treatment regime [41].

#### 2.1.2 Non‐electrophoretic separation techniques

Chromatography is an essential technique for separating, identifying, and purifying the components of a mixture for quantification and qualification. In this method, proteins are purified based on characteristics such as molecular size and shape, total load, hydrophobic groups present on the surface, and the capacity to bind to the stationary phase. Many of these methods are amenable to high‐throughput automation, integration with MS instruments, and achieve high yields and purity with excellent reproducibility [42].

High performance liquid chromatography (HPLC) and fast protein liquid chromatography (FPLC) are two widely used liquid chromatographic methods. HPLC is used with the aim to obtain a complete protein recovery, and protein separation is based on some specific protein properties (hydrophobicity, surface charges, and specific amino acid sequences). This method pumps a liquid solvent containing the sample mixture through a column filled with a solid adsorbent material. For this purpose, high‐pressure procedures (up to 400 bar) are required with organic solvents and these methods are commonly restricted to relatively low loading of the sample. HPLC of biomolecules is very complicated and susceptible to conditions, being unable to resist the high temperatures, high pressures, or the solvents employed. Therefore, biomolecule separation demands an alternative strategy. Therefore, FPLC has been designed to supply a more biocompatible separation of biomolecules with high resolution. FPLC utilizes a relatively low backpressure to drive the high flow rates to separations performed; therefore, it is appropriate for large biomolecule purification like proteins [43]. In addition, FPLC has an approximately 30 times cheaper price per test than HPLC. The FPLC column cost itself is about 10 times less than that of the HPLC column [44].

**FIGURE 2:**
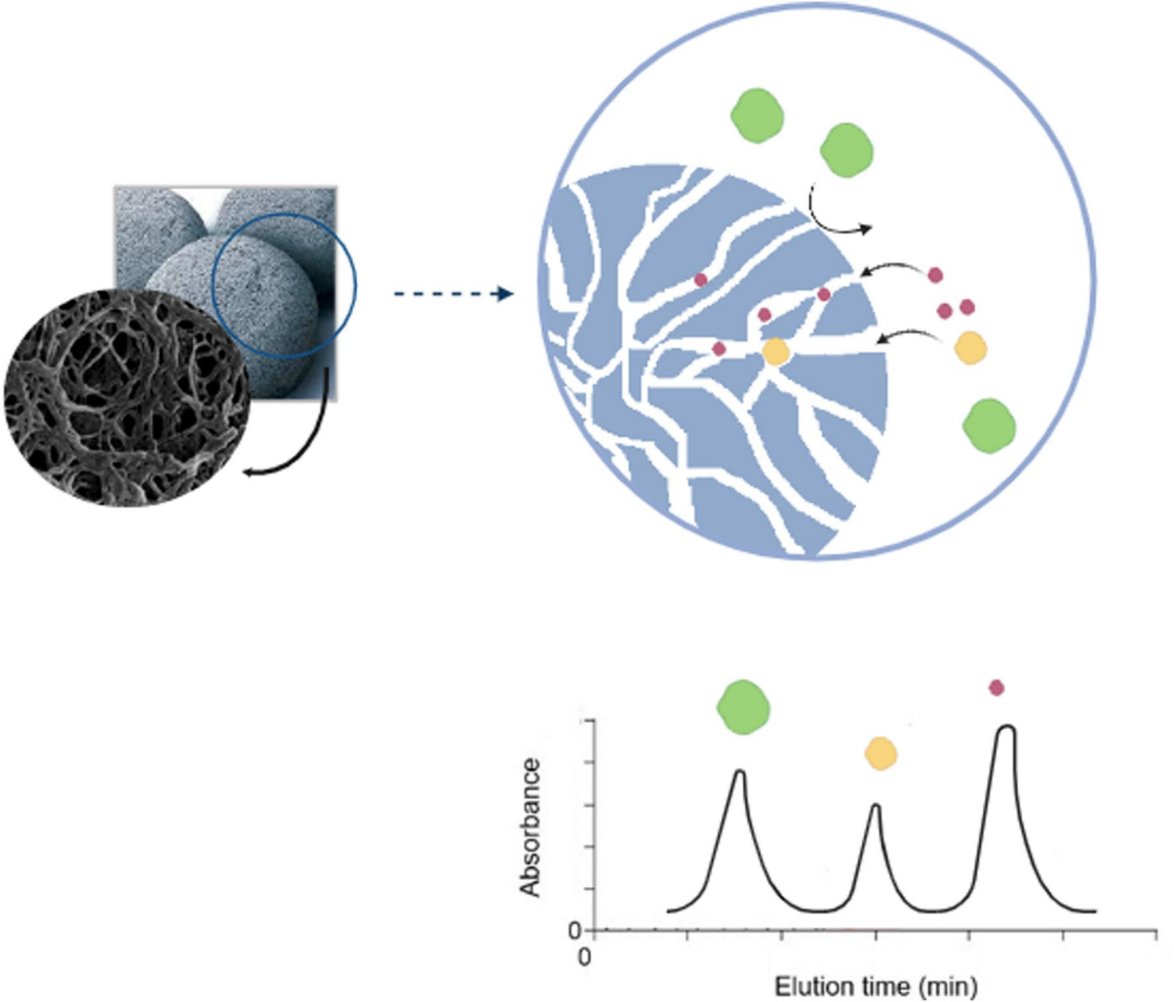
SEC principle. Small molecules (purple) enter pores in the matrix, so travel slowly and are eluted later. Medium molecules (orange) enter some pores, and large molecules (green) enter few pores in the gel, and so travel rapidly.

A number of liquid chromatographic techniques are used to separate proteins by size, namely size exclusion chromatography (SEC) and gel filtration chromatography (GFC); both of these use the same principle [45]. SEC is a size partitioning chromatography that performs the entire separation of large molecules from much smaller ones. First, the sample molecules are dissolved in an appropriate solvent (completely dissolve the studied components), and then they are passed into a gel matrix in a column. Smaller particles flow more into the pores of the matrix, while larger molecules do not enter the pores and travel faster than other molecules (Figure 2) [46]. Depending on the characteristics of the analytes, a class of liquid chromatographic techniques, GFC is presented. GFC uses columns including functionalized beads with polar groups to separate water‐soluble compounds by the utilization of aqueous mobile phases. It separates macromolecules by size within the included volume of the stationary phase and has relatively high chromatographic resolution. These are the methods best suited to highly concentrated samples, are simple to implement, and can be achieved using relatively simple equipment.

In ion chromatography, the separations of both anions and cations are done based on ionic interactions among ionic analytes, eluent, and functional groups organized on the stationary phase, and also based on the pH (chromatofocusing). The latter can be employed to separate aqueous ppm amounts of anions in the sample (0.5–150 ppm per anion at 100 μL injection sample). It is typically used in bind‐elute mode or flow‐through mode. The bind‐elute mode can be utilized to concentrate initially dilute samples. In addition, it is used to recover a target protein whereas the contaminants are bound with minimal manipulation to sample [47]. It is a helpful and conventional system due to its high resolving capability, soft separation conditions (for unstable protein), versatility, and widespread utilization in industry, and it is relatively cheap to operate compared to hydrophobic systems [48].

Multiple dimensional liquid chromatographies can be performed, based on the combination of the different liquid‐phase separation, such as chromatofocusing and nonporous reverse phase column chromatography. This provides a high throughput and reproducible separation system for the separation of complex mixtures in mammalian cells. However, the quantitative computation of protein in a sample is a challenge [49]. Capillary liquid chromatography (LC) connecting to a microfluidic device is a hybrid multidimensional separation system based on the coupling of the various practical components, limiting the quantity of sample, reducing the quantity of solvent used, and getting rapid and accurate outcome. Amplification of the traditional column peaks and their sensitivity are further advantages [50].

### 2.2 Sample analysis and data acquisition in biomarker development

Several methods and tools can be used to attain protein information. There are several principal proteomics techniques to identify proteins, including enzyme‐linked immunosorbent assay (ELISA), WB, Edman sequencing, and MS [51]. ELISA, a sensitive and precise immunological assay, is commonly employed to analyze the quantification of particular proteins [15]. Its workflow will be described in Section 2.4.2. Western blot is also an immunological assay (affinity antibody‐based technique) that detects specific proteins that at first are separated by electrophoresis and then transferred to the nitrocellulose membrane. Despite its advantages in protein identification, this method is laborious and time‐consuming, and cannot provide quantitative measurement. In addition, affinity antibodies usually have high production costs and are prone to irreversible denaturation [52]. Edman sequencing was the earliest method used for protein identification by fluorescently labeling [53].

**FIGURE 3:**
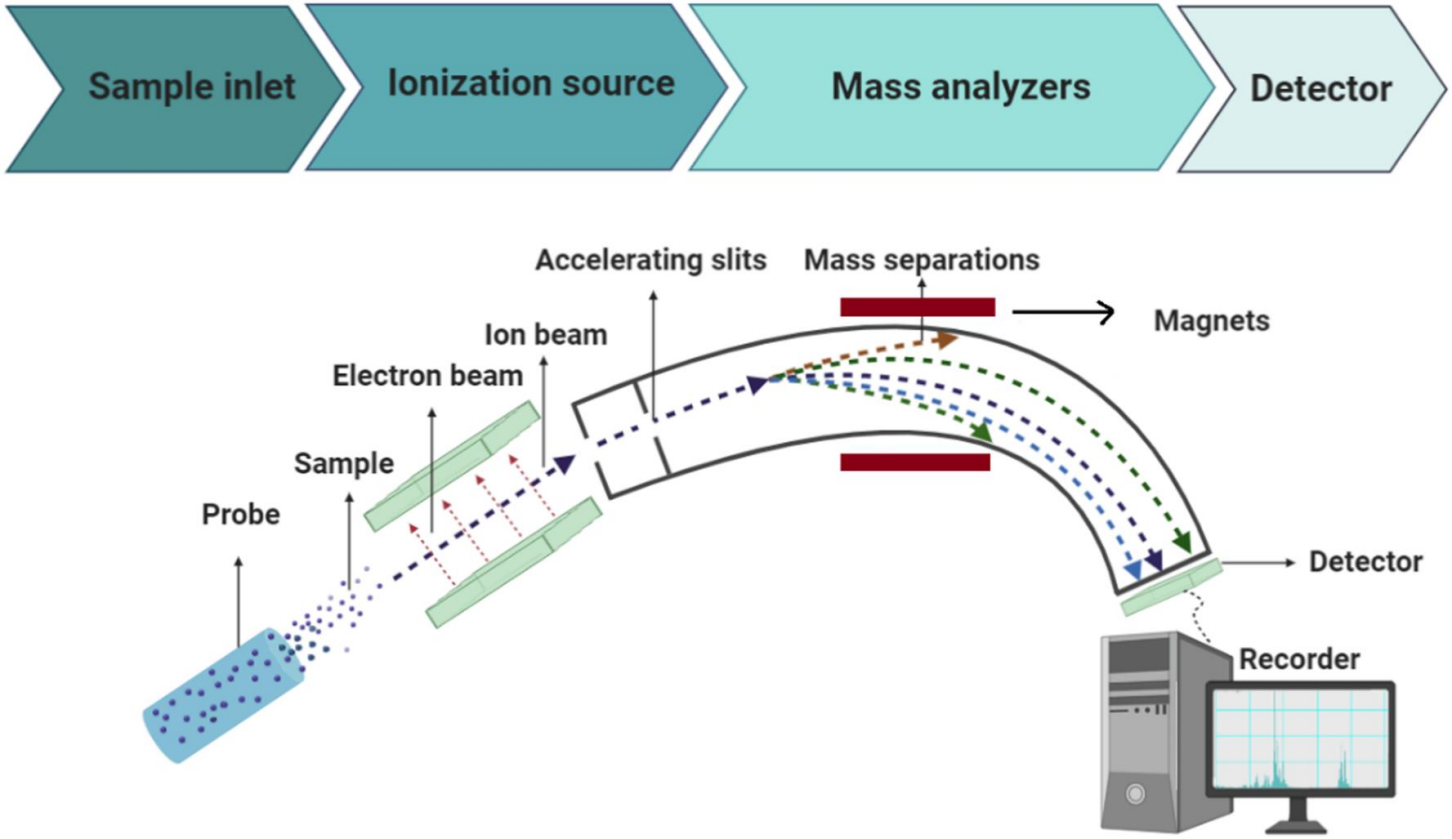
A basic diagram of the mass spectrometer and different parts of the device.

Finally, MS is a powerful and precise technique with prominent sensitivity for peptide detection that enables determination of the molecular masses and fragmentation of proteins and peptides. It also gives sequence information for protein identification, and can identify and localize post‐translational or other covalent modifications [54]. The mass spectrometer is the typical identification approach, generally consisting of a sample inlet to introduce the compound, a direct probe for inserting the sample, an ion source to generate ions of the primary protein molecules of the sample, the mass analyzer to divide the different ions, and a detector to compute the ions appearing [55]. The most common mass analyzers employed for precise protein quantification are quadrupole, Orbitrap, and ToF [56]. A basic diagram of the mass spectrometer is displayed in Figure 3.

A tandem mass spectrometry (TANDEM MS, also named MS/MS), is a kind of MS employing two or more mass spectrometers connected, or a mass spectrometer with multiple analyzers organized successively [57]. TANDEM MS with a collision cell‐facilitating ion fragmentations between them enables further ‘purification’ by choosing target precursor molecular ions for other analysis and characterization [58]. In TANDEM MS investigations, multiple MS dissociation procedures can be operated to cause structural fragmentation of precursor ions [59]. There are several types of MS/MS fragmentation processes, such as electron‐transfer dissociation (ETD), electron‐capture dissociation (ECD), and collision‐induced dissociation (CID) [60]. A common and straightforward mechanism to implement the process is CID, which employs collisions of the fast‐moving peptide ions with neutral inert gas [61]. Hence, in tandem MS, at first, the peptides are separated by MS spectrum, and then specific peptides with particular mass are selected for collision‐induced dissociation (CID) in a collision cell. A dissociation cell contains a small amount of inert gas that is able to fragment the precursor ions with sufficient energy to break the peptide bonds. The operating energy is optimized for inefficient fragmentation to create the ions corresponding to peptides of differing lengths [62]. In this strategy, the isopeptide bonds are also exposed to fragmentation and their modification may not be detected, therefore more delicate fragmentation techniques are required. ETD is designed as a fragmentation process to keep more labile modifications, such as phosphorylation, methylation, acetylation, glycosylation, nitrosylation, and sulfation [63]. Specifically, beam‐type CID sometimes referred to as higher‐energy C‐trap dissociation (HCD), is a CID technique specific to the orbitrap mass spectrometer in which fragmentation takes place outside the trap. In HCD, the ions pass through the C‐trap and enter the HCD cell, an added multipole collision cell, where fragmentation takes place [64]. Compared with conventional ion trap CID, HCD fragmentation with orbitrap detection does not have a cutoff for low mass, even though it has high‐resolution ion detection, and high‐quality tandem MS spectrum [65]. Therefore, to have valid data interpretation, it is critical to understand the device setup and the effects of the fragmentation process on results.

Briefly, CID is commonly recommended for peptide fragmentation because it can analyze the phosphoribose existence by fast scan rate and the partial loss of the PTM. However, HCD and ETD are valuable for specific peptides that are quite large, highly charged, and cannot be typically fragmented by CID [66]. Due to the critical role of MS in protein biomarker identification and quantification, in the following section, 2.2.1, various MS‐based strategies in protein biomarker development and data collection are studied.

#### 2.2.1 Types of the MS‐based quantification in protein biomarker development

##### Based on the study aims

There are two main strategies in the MS‐based proteomics analysis: bottom‐up and top‐down. Overall, in the bottom‐up workflow, proteins undergo enzymatic digestion by a protease, and the sizes of proteins are reduced from hundreds of amino acids to peptides with six to 30 amino acids. Therefore, at first, the specific protein is separated by the LC, then it is fragmented by enzymatic digestion, and then finally coupled to MS [67, 68]. When the bottom‐up methods are employed on the complex protein sample (such as proteome), the untargeted procedure is termed as shotgun proteomics [69]. Which, the entire proteome is digested with a protease to produce a mixture of peptides, and then the peptide mixture is loaded onto a chromatography column, and the peptides are separated. The peptides are ionized subsequently and identified by MS. By contrast, in the top‐down workflow proteins are isolated from their natural source without enzymatic digestion. Therefore, the proteins are ionized in the first step and then the fragmented ions are produced from the intact protein ions, which cause to create complicated MS/MS spectra of proteomes with the hard interpretation that requires experienced interpretation [70, 71]. The commonest method employed in candidate biomarker identification is the bottom‐up strategy, in which DDA, DIA, and targeting methods are the three main acquisition modes [72].

MS‐based analysis has two basic modes for data collection, including data‐dependent acquisition (DDA) and data‐independent acquisition (DIA). In DDA, also known as information‐dependent acquisition mode, the MS device selects a subset of the most abundant ions in the first stage of the MS/MS (MS1), and afterward, they become fragmented and are identified in the second step of MS/MS (MS2). Each MS2 scan can be analyzed with a database search algorithm (Figure 3). While one of the advantages of DDA is selectivity, when many types of peptides appear in a single MS1 scan, the irreproducibility and inaccuracy are significant in DDA, so in the MS2 scan of DDA, only the most abundant peptides are scanned and missed the remains, which restricts its application in high‐throughput analyses [73, 74].

**FIGURE 4:**
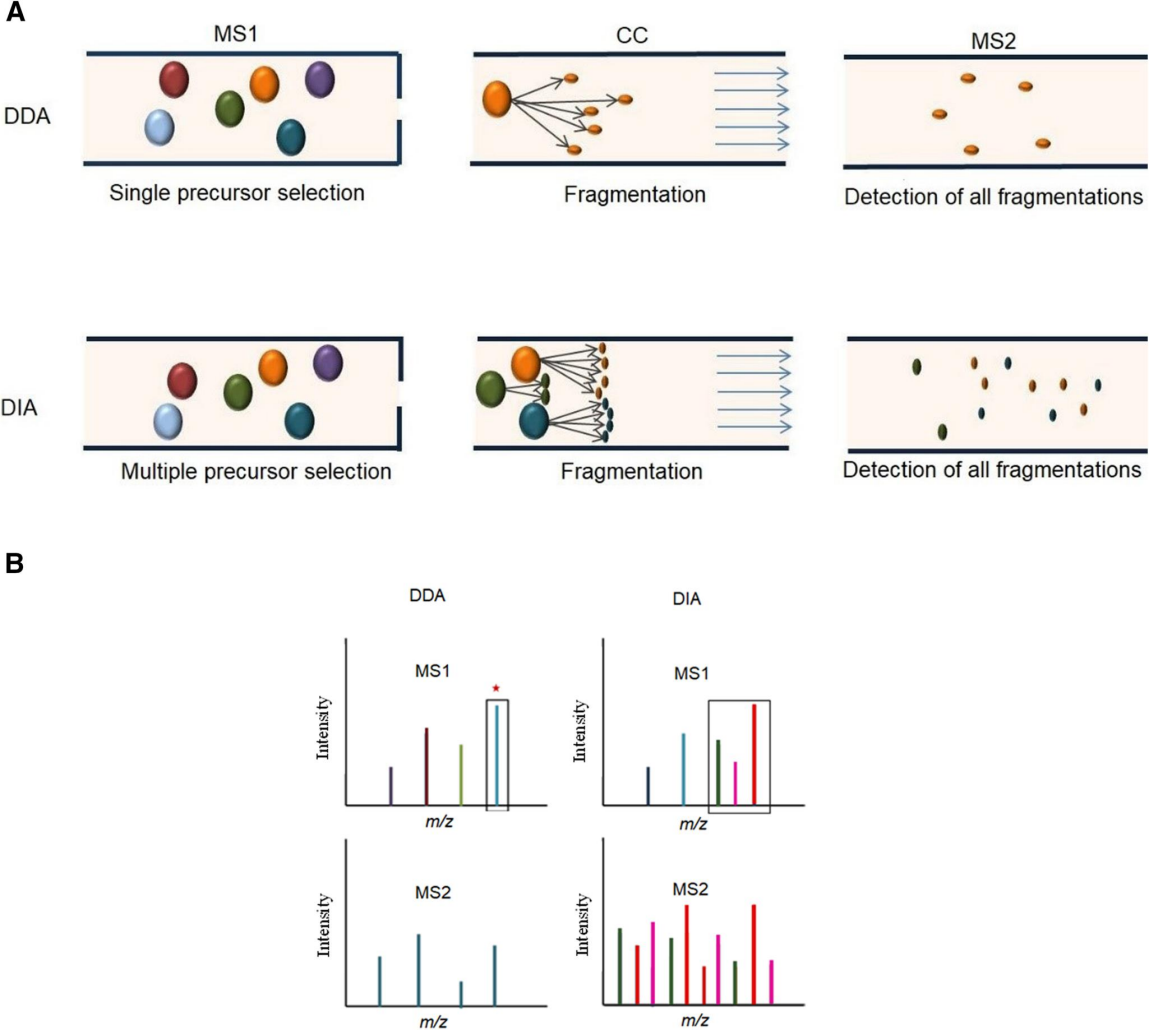
(A) Isolation, fragmentation, and detection of fragmented ions. (B) MS1 and MS2 spectra in DDA and DIA data acquisition modes. In DDA, at first, the most intense precursor ions from the MS1 scan is selected (blue peak) and only these selected peptides are fragmented for tandem MS analysis. In DIA, all ions within a defined range of *m/z* window are fragmented and analyzed (pink, green, and red).

The DIA mode of MS has been lately developed as a promising choice for quantitative proteomics analysis [75]. The DIA procedure is based on the fragmentation of every single peptide in a sample and focusing on a narrow mass window of precursor ions in a range of relevant mass‐to‐charge ratio (*m/z*) values and then scanning all the fragments (Figure 4) [76]. The DIA strategy has slightly higher accuracy and high reproducibility than the DDA, and is widely used because a large amount of protein can be identified and quantified in a single run. Sequential window acquisition of all theoretical fragment‐ion spectra (SWATH) is a specific model within the DIA strategy in which all precursor ions with a pre‐defined *m/z* range are fragmented [77, 78]. The SWATH‐MS method is accomplished by a DIA procedure followed by a novel targeted data extraction process [79], and has the advantage of quantification with high consistency, covering thousands of proteins. It is an ideal technique for a large number of specimens that require a quantification with precision and reproducibility for the main fraction of the expressed proteins or peptides, such as in biomarker investigations [80]. In 2020, Maine et al. described a new method that employed SWATH mass spectra in the multi‐dataset joint analysis to display the first stage of gastric cancer plasma biomarkers, which detects 37 proteins with high expression profiles and 21 proteins with low expression profiles in the plasma of gastric cancer patients [81]. In another study in 2020, Singh et al. presented SWATH‐LC‐MS/MS analysis to report quantitative proteomic comparisons involving the transition from androgen‐dependent prostate cancer to androgen‐independent prostate cancer by induction of exogenous TGF‐β. The effect of known proteins on the long‐term survival of cases was also investigated [82].

As previously described, DIA and DDA proteomics (as global proteomics) are two procedures for biomarkers discovery, while an additional validation phase is also required as a targeted approach to enhancing the accuracy, sensitivity, and quantification of a particular protein. The essential methods in targeted MS techniques are multiple reaction monitoring (MRM) and parallel reaction monitoring (PRM), which present the most appropriate interpretation for absolute quantification when employing internal standards. These methods will be described in detail in Section 2.4.2.

##### Based on provided information and methodology

Another classification of MS quantifications is based on protein labeling that involves label‐based and label‐free approaches (to determine the absolute concentration of distinct proteins within a sample). These are generally summarized in terms of comparison of whole proteomes, terms of the relative amounts of proteins or in terms of absolute quantification, to determine the absolute concentration of distinct proteins within a sample.

The first standard technique for quantification of candidate biomarkers relies on stable isotope labeling analysis. Label‐based methods compare the two or more experimental samples based on a specific difference in the mass, which is due to labeling them with alternative differential mass isotope/isobar tags. Label‐based approaches employ specialized isotope/isobar tags with particular groups that label proteins and peptides chemically, metabolically, or enzymatically [83]. Chemical labeling employs chemically synthesized tags by integrating various isotopes and isobars to apply mass shifts within the proteins. For example, isotope‐coded affinity tags (ICAT) as essential tags in protein labeling consist of three available elements, that is, iodoacetyl group that binds to thiol‐specific groups, a linker that induces mass differences, and biotin utilized for affinity purification. While, in the metabolic labeling technique, the isotopic amino acids and components is biologically integrated via cell culture and dietary food in plants and animals, respectively. Enzymatic labeling induces the substitution of natural (16O) and isotopic oxygen (18O) in the carboxyl groups of amino acids in proteins [84]. Several major strategies are presented to insert the mass tag into the protein or peptide, such as chemical modification after protein expression, labeling metabolically during in vivo expression, isotopically labeled references by directly synthesizing, or label insertion during proteolytic digestion of the proteins. The quantification of protein can also be in relative terms (protein quantitative ratio or fold distinction relative to specific protein or whole proteome of different samples) or in absolute terms (measuring the protein concentration in sample) [85]. Some possible limitations exist for most label‐based quantification approaches, such as the complexity of the preparation of the sample, the prerequisite for high specimen concentration, and insufficient labeling. There are several steps in the preparation of the sample, high prices, and restricted sample size studied within one investigation [58]. Utilizing the MS1 spectrum is another limitation [61]. In the larger sample size because of the overlapping peaks and similar characterization of precursor ions, the MS1 spectrum is difficult to investigate [61]. So, this restricts the number of samples that can be investigated in a single analysis [60]. Another restriction is the limited number of ions that can be collected in the generally employed analyzer, the Orbitrap, with high resolution [62, 63]. If the peptides are less abundant, fewer ions will be produced, so the accuracy will be significantly reduced. This restriction has been partially removed by ion‐mobility separation or BoxCar [60]. In addition, quantification data can be also acquired from the MS2 fragment ion spectra. In this case, two or more independent spectra are developed for investigation. However, isobaric label quantification can be confused due to co‐isolated peptides, which form reporter tags, and if superimposed on the reporter tags from the selected precursor ion, provides an incorrect connection between peptide abundance and individuality [64]. The double isolation method, MS3, was proposed to handle this concern [65].

Regarding the essential role of the differentially expressed intact N‐glycopeptides in cancers and other pathological processes, Wang et al. reported a method for the relative quantification of intact N‐glycopeptides using stable isotopic diethyl labeling of amino groups. A dynamic range of linear quantification up to 50‐fold was obtained. Hence, stable isotopic diethyl labeling can be used in a quantitative study of any unusual glycosylation at the level of an intact glycopeptide [86]. In 2020, Zhou et al. obtained candidate protein biomarkers for early‐stage gastric cancer screening through MS and bioinformatics technology. By plasma proteomics applying LC‐MS/MS combined with tandem mass tag labeling, they identified proteins that could help to distinguish between early‐stage gastric cancer and healthy controls [87].

The second quantification technique is label‐free DDA proteomics. As mentioned earlier, and in contrast to labeled‐based methods, both relative and absolute label‐free protein quantification techniques are evaluated by precursor ion peaks (MS1) obtained from tryptic digestion or spectral counts, and can be connected to peptide fractionation techniques to develop more proteome coverage [80]. The advantages of label‐free quantification (LFQ) over label‐based methods are sample preparation phases, avoiding extra cost, proteome coverage, and greater dynamic range. Moreover, it is possible to use LFQ for many samples, from hundreds to even thousands [88]. However, there are many limitations to LFQ. The main limitations are the number of missing values, especially for low concentration peptides and proteins—this number is higher than all quantitative proteomic technologies—and also the requirement of multiple runs, which decreases accuracy and throughput [89].

In 2018, Sandow et al. reported the first recruitment of LFQ and LFQ‐PRM to detect ovarian cancer biomarkers and introduced methods needed for further research to assess biomarkers of ovarian cancer‐specific disease. They employed LFQ, followed by targeted PRM, to discover and validate ovarian cancer‐related protein biomarker candidates in urine samples [90]. In 2020, Oliveira et al. utilized an LFQ peptidome approach to identify variants in serum levels of peptides to identify serum‐type peptides that could distinguish gastric adenocarcinoma patients from controls. Their laboratory outcomes explain that the serum levels of peptides are significantly modified in the physiopathology of gastric adenocarcinoma [91].

### 2.3 Database utilization

As described above, utilizing omics technologies such as proteomics have been confirmed to be an effective strategy to recognize biomarkers. It is noteworthy that the application of this technology along with bioinformatics tools is required for the identification of molecular targets. Therefore, in the last step of identification and quantification of protein biomarkers, after the collection of the protein biomarker fragmentation spectra from MS, the peptide sequences need to be determined. Two different strategies can be employed for this purpose: exploring the fragmentation spectra databases, and de novo peptide sequencing. In first strategy, a peptide spectrum score for all peptide MS spectra generated is calculated and compared to theoretical database spectra of in silico fragmentation of each expressed or possible protein sequence [92]. The peptide with the highest score can be accepted as a candidate. There are many database search engines. MaxQuant [93], SEQUEST [94], pFind [95], and TopPIC [96] are some examples of peptide and protein identification databases. In de novo peptide sequencing, the peptide sequence is only discovered by fragmentation spectra information and its method aspects. De novo sequencing does not need any reference databases, so it has a strong advantage in identification of novel protein sequences. Over the past decades, many de novo sequencing algorithms for shotgun proteomics have been proposed, such as PEAKS [97], PepNovo [98], DeepNovo [99], pNovo 3 [100], UniNovo [101]. Once the identification of the peptide is finished, the next step is to determine the peptide sequences within the primary proteins. This method is described as protein inference. The excess level of biomarker over the whole proteome in a specimen can be obtained by a quantitative proteomics study that will be described.

### 2.4 Discovery, verification, and validation of the biomarker

**FIGURE 5:**
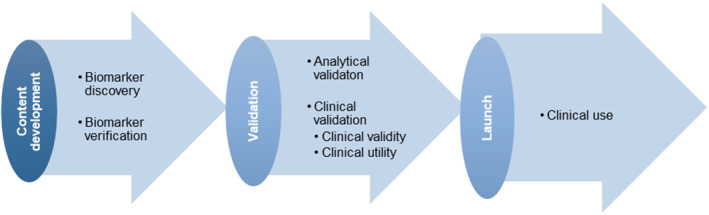
The pipeline of biomarker development.

The development of protein biomarkers has always been critical to the clinical study community. Since 2019 alone, there have been more than 50,000 publications containing the term “protein biomarkers” in PubMed. The biomarker development pathway is divided into three phases: biomarker discovery, verification, and validation (Figure 5) [102].

Biomarker investigation for any disease, particularly cancer, must be evaluated carefully before starting any treatment. A biomarker discovery study should consist of the following steps: determination of the disease type, the number of patients and controls, choice of patients (age, sex, etc.), type of samples, etc. [103]. As previously mentioned, optimizing the number of samples is a requirement. In the first phase of biomarker development, biomarker discovery, a minimal number of samples is required to provide sufficient statistical requirements; the number should be adequate for the guarantee of reliable results without high false positives or false negatives rates. However, most patients or control samples cause ethical, efficiency, and cost problems. Ideally, the optimal sample size should be chosen utilizing calculations based on previous knowledge or a statistical theory [104]. The conventional strategy for optimal sample size determination is power calculation [105]. Although, this method has some problems that occur because of the assumption of a high level of correlation between data points, and of maximizing the power to separate classes; classification algorithms aim to maximize prediction accuracy [106]. Various approaches have been suggested but there is still no method that overcomes all of the limitations. In the following, each of the biomarker discovery methods has been explained and the appropriate sample size for each phase of biomarker discovery has been given.

#### 2.4.1 Biomarker discovery

The protein biomarker discovery stage involves measuring many proteins in different samples and is primarily based on in‐depth, untargeted proteomic analysis to provide an initial list of proteins that may be involved in disease progression [107]. MS plays a central role in the identification and quantification of candidate biomarkers [108]. In this phase any type of specimen can be employed, for example, a mouse model, cell line, or a variety of human physiological specimens to develop a binary comparison between diseased and healthy tissues (as control), without any ‘contamination’ by different diseases or other conditions [109]. One example is in the identification of the protein with a distinct expression profile in inflammatory bowel patients but not in normal controls [110]. When the identification of a specific biomarker in a particular subtype of the disease is the aim, various samples of a disorder can be used as a case or control [111]. Because of the cost, logistics, and relatively low throughput of the discovery stage, this phase often uses a limited sample size (typically 10) [109, 112, 113, 114, 115]. The low sample size and analytical diversity can lead to a high false‐positive rate. Therefore, after the identification of candidate proteins, verification and validation are necessary and accomplished with a higher number of samples. In the biomarker verification phase, to select the highly specific and sensitive biomarkers, several hundred samples are evaluated. In addition, in this phase, the absolute amount of each peptide is measured instead of their relative quantification [116].

#### 2.4.2 Biomarker verification

As mentioned in the previous section, candidate biomarkers identified in the discovery step must be confirmed by many samples. The verification phase calculates the relative concentrations of the candidate biomarkers in a reasonable number of patient specimens [107]. In this phase, the analysis is performed on a larger sample size with a wider range of patients and with normal specimens to capture more variation in the population [109]. The cohorts of specimens should be extended to obtain a statistically powerful measure of the potential biomarker; for example, there should be samples acquired from disease‐affected and healthy donors in addition to those having a similar disease. For example, samples should be from males and females with a broad range of age, pre‐ and post‐menopausal women, etc. [115]. The challenge is the development of a quick‐targeted test that is able to analyze as many identified candidates as possible in hundreds to even thousands of samples. A biomarker candidate verification step can overcome this and ensure that the most putative biomarkers identified in the first phase are delivered to the expensive validation step. Currently, ELISA, MRM [117], PRM [118], in combination with stable isotope‐labeled internal standards, have been extensively examined as options for biomarker verification [116].

**FIGURE 6:**
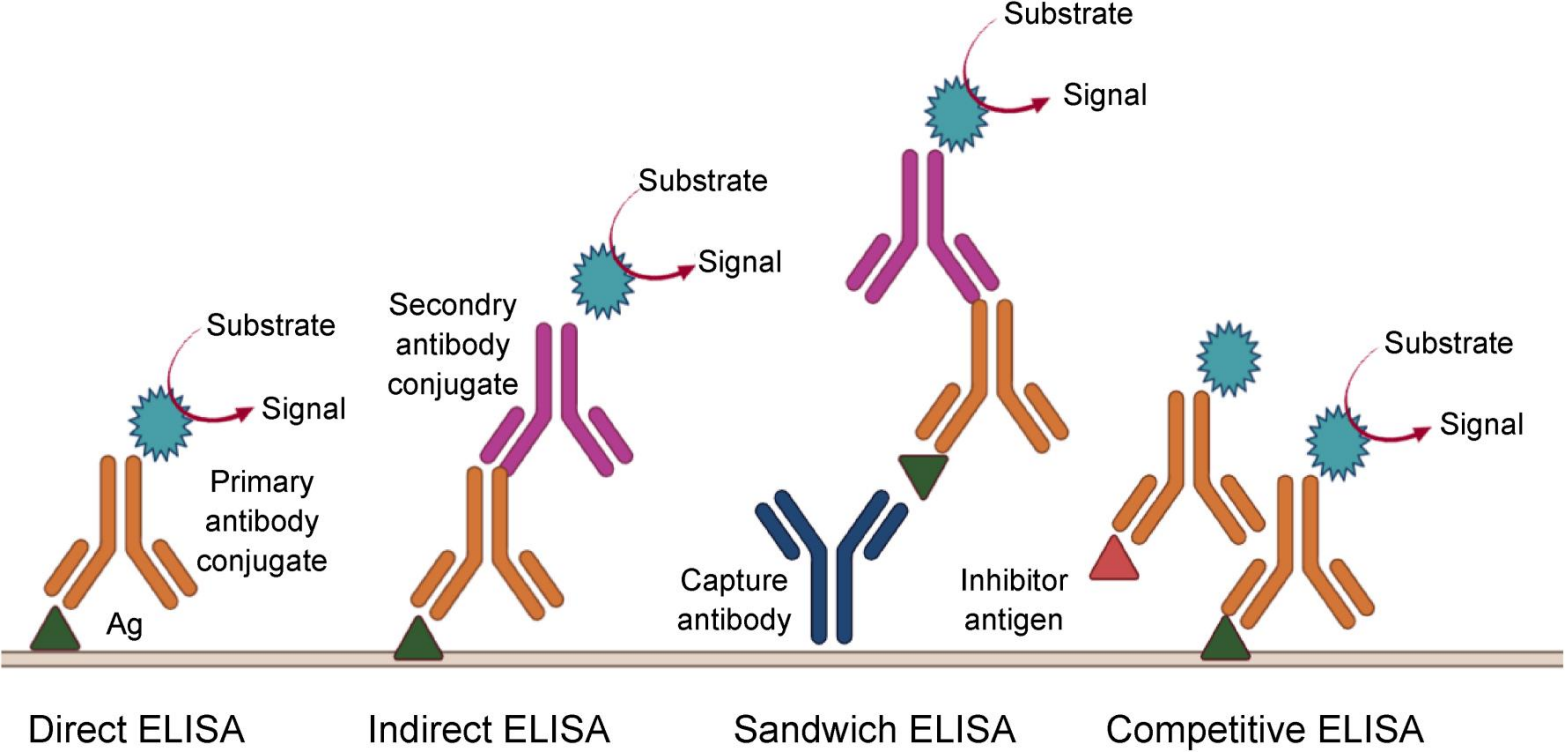
Different types of ELISA test and mechanism of specific molecule detection.

ELISA is a sensitive and high throughput immunological method that is employed for the identification and quantification of the biomolecules, such as proteins, antibodies, glycoproteins, antigens, and hormones, in biomarker verification [119] based on the antibodies‐antigens complex that provides measurable outcomes. ELISA encompasses four essential steps: (1) coating (with antigen or antibody), (2) blocking (commonly by adding the bovine serum albumin), (3) detection, and (4) reading. In step 3, adding a substrate that produces a color causes detection [120]. Based on the types of analytes and antibodies conjugation on the surface and their production signals, ELISA is categorized into various individual types of assays, including direct, indirect, sandwich, and competitive (Figure 6). Ourradi et al. developed the ELISA immunoassay for quantitative measurement of two novel biomarkers, C3f and V65, which seem to be osteoarthritis‐specific biomarkers and have the potential for early detection of disease [121].

MRM (also known as selective reaction monitoring—SRM) has essential roles in metabolic and pharmacological studies, including drug analysis, and human body fluids proteins [116]. These instruments possess the power to choose a parent peptide ion (MS1) of the desired protein based on *m/z* to promote CID, and then keep track of select fragment ions (MS2) based on *m/z* [122]. There are two advantages to the MRM technique. The first one is high sensitivity and selectivity, and label‐free operation with no need for antibodies, which makes the preparative workflow streamlined. The second is the ability to quantify many peptides simultaneously, which increases throughput. In a recent study, Chi et al. applied MRM and SISCAPA‐MRM, for quantification of 30 potential selected biomarkers of oral cancer in both plasma and saliva samples, which ultimately have been discovered and/or verified [123]. In another study, Sjödin et al. used a technique that measured ubiquitin concentration, specifically by coupling SPE and PRM–MS, to evaluate cerebrospinal fluid (CSF) ubiquitin as a biomarker for neurodegenerative dysfunctions [124].

PRM is an alternative method for quantification of specific molecules, and has high determination and accuracy [125]. PRM offers an entire scan of any transfer by a precursor ion with parallel monitoring of each segment from the ion. One of the properties of this method is its straightforwardness, so it is appropriate for repeated consideration of pre‐determined molecules, such as cancer biomarkers [126]. For example, in 2022, Bao et al. identified and verified COPA as a potential prognostic biomarker by using MS‐based quantitative proteomics and targeted PRM in freshly frozen cervical cancer tissue samples [127]. Research employing SRM and PRM has confirmed that both targeted approaches have relative sensitivity with related linearity, dynamic amplitude, accuracy, and reproducibility for protein quantification [128, 129].

#### 2.4.3 Biomarker validation

After the detection of a small number of biomarkers in the verification phase, the validation phase analyzes the external reproducibility independently of the cohort. The biomarker validation phase requires a higher number of clinical samples (e.g., >1000) relative to the discovery stage (e.g., <100) [116]. To date, ELISA is the most conventional technique for biomarker validation, and it can prognosis various samples simultaneously [109]. There are two types of validation: analytical validation and clinical validation.

##### Analytical validation

Analytical validation involves the study of biomarker efficiency to confirm that a test is reproducible with adequate sensitivity and specificity for the required purpose [130]. In clinical validation, the degree of association and reliability of the result, and clinical phenotype or the desired effect are demonstrated [131]. Several recent analytical validation studies of cancer biomarkers that illustrated this workflow will be reviewed.

Park et al. appropriated a proteomics strategy to identify 90 protein biomarker candidates and validate them using both MS and ELISA methods [132]. In 2020, Hurley et al. evaluated five paraneoplastic antigens, along with three tumor‐associated antigens, in serum samples from patients with high‐grade ovarian cancer. In their study, validation screening with ELISA and WB was performed using two independent sample sets, validation I, which included 164 samples, and validation II, including 150 samples [133]. In 2020, Zheng et al. reported DIA‐MS‐based utilization in liquid biopsies. They examined EV protein/phosphoprotein biomarkers in the fluid biopsy of colorectal cancer cases and identified FN1, S100A9, FGA, and HP, with significant variation in protein phosphorylation and expression. Their results confirmed that FGA + crEV have almost 100% sensitivity in the diagnosis of colorectal cancer and 65% sensitivity in the early diagnosis of adenoma patients. They ultimately validated the DIA‐MS quantification of FGA and crEVs among three groups by PRM‐MS [134]. The essential phases and different methods that are frequently used in analytical validation are represented in Table 2.

**TABLE 2:** Essential methods in the main phases of biomarker development.

Phase	Typical number of specimens	Techniques	Refs.
Biomarker discovery	10 specimens	Mass spectroscopy‐based gel electrophoresis; Mass spectroscopy‐based capillary electrophoresis‐MS; Mass spectroscopy‐based gas chromatography‐MS; Mass spectroscopy‐based liquid chromatography; Offline NMR with liquid chromatography; Enzyme‐linked immunosorbent assay; NMR with mass spectrometry; Immunohistochemistry blotting	[135, 136, 137]
Verification	100 specimens	Enzyme‐linked immunosorbent assay; Multiple reaction monitoring; Parallel reaction monitoring; Single reaction monitoring	[116, 138]
Validation	>100 specimens	Enzyme‐linked immunosorbent assay; Multiple reaction monitoring; Parallel reaction monitoring; Surface plasmon resonance; Western blotting	[132, 133, 134, 139, 140, 141, 142]
Clinical validation	1000 specimens	Enzyme‐linked immunosorbent assay; Enzyme‐linked immunospot; Flowcytometry	[143]

##### Clinical validation

A challenge in clinical assay development is the propagation of a biomarker identified in the laboratory as a promising candidate into a format that matches clinical utility. Clinical validation, as a last step in the evolution of biomarkers, must confirm the relationship between the biomarker and the endpoint of interest, and demonstrate clinical validity and clinical utility. This requires more time than the analytical method, and also checks the degree of association and reliability of the test result with the clinical phenotype or the desired result [144]. Cohort studies and the type of biomarker used in the distinctive cancer screening should be considered in the clinical validation phase for acceptance criteria [145]. Different mass spectrometric methods may be utilized for the various stages of the biomarker pipeline, but in the conventional clinical setting, a simple reproducible assay that does not require highly technical and specialized expertise and equipment is preferred [113].

## 3 DISCUSSION

Protein research has attracted much interest in recent years. One of the most important aspects of the success is the development of powerful novel techniques for the separation and identification of proteins. Proteomic workflows rely heavily on MS and recent advancement includes powerful new technology (such as MALDI) to increase its sensitivity, accuracy, and throughput in biomarkers discovery. In addition, it can be integrated into multiple separation and preparation methods to determine the target protein biomarker with high accuracy and yields [146]. Protein biomarker verification is also an essential phase that can use ELISA, MRM, PRM, and SRM in combination with stable isotope‐labeled internal standards. ELISA is the most conventional technique to simultaneously process various samples for prognosis biomarker development. Recently, analysis of protein biomarkers based on DIA‐based untargeted methods and PRM‐based targeted validation have improved the sensitivity and quantitative precision, and selectivity of proteomics research [38, 90, 91, 92]. Moreover, joining various proteomics technologies for orthogonally validating a candidate biomarker is an essential strategy [147]. Unlike advancements in MS‐based approaches and comprehensive investigation, the discovery and validation phases of the protein biomarkers to screen and diagnose are restricted by the dynamic complexity of the samples. In addition, a further challenge for proteomics analysis is the absence of an effective study design (in particular, relating to small sample size) that causes doubtful results that are not reproducible and have poor statistical power. So currently, operated protein biomarkers have low sensitivities and selectivity for use in clinical research, and it is now well established that a single biomarker is not sufficient but that a panel of proteins is required for diagnosis. Another problem is the validation of new biomarkers and accessing data based on large groups of patients. However, the collection of data achieved in various laboratories can ultimately contribute to the validation procedure via meta‐analyses [51]. Ultimately, for screening goals, multicentered broad analyses and meta‐analyses should be performed to enhance precision and sensitivity.

## 4 CONCLUSION

In conclusion, biomarkers continue to play a key role in disease diagnosis, drug discovery, and patient care monitoring. In recent years, advancements in the capabilities and sensitivity of proteomics techniques have led to the identification of new protein‐based biomarkers.

This review described proteomics workflows in biomarker development and analytical scheduling, from the preparation of different samples to purification and data analysis. It scans the field of MS‐based proteomics as a technique, especially in cancer biomarker discovery and explores in the verification and validation phase of novel biomarkers and their various methods. This review also clarifies how MS has become an essential method for biomarker development but highlights that it is most efficient if applied alongside other approaches.

## AUTHOR CONTRIBUTIONS

**Mahsa Babaei**: Investigation; methodology; writing original draft. **Soheila Kashanian**: Planning and supervision. **Huang‐Teck Lee**: Validation, visualization, review, and editing. **Frances Harding**: Validation, visualization, review, and editing. All authors reviewed the results and approved the final version of the manuscript.

## CONFLICT OF INTEREST STATEMENT

The authors Mahsa Babaei, Soheila Kashanian, Huang‐Teck Lee, and Frances Harding declare that they have no conflict of interest.

## ETHICS STATEMENT

This is a review article and does not involve any research related to human or animal subjects.
